# Differential pathogenicity of two different recombinant PVY^NTN ^isolates in *Physalis floridana *is likely determined by the coat protein gene

**DOI:** 10.1186/1743-422X-8-207

**Published:** 2011-05-07

**Authors:** Xinxi Hu, Xianzhou Nie, Changzheng He, Xingyao Xiong

**Affiliations:** 1 Hunan Provincial Key Laboratory of Crop Germplasm Innovation and Utilization, Hunan Provincial Engineering Research Center for Potatoes, College of Horticulture and Landscape, Hunan Agricultural University, Changsha, Hunan 410128, China; 2 Potato Research Centre, Agriculture and Agri-Food Canada, P.O. Box 20280, 850 Lincoln Road, Fredericton, New Brunswick, E3B 4Z7, Canada

## Abstract

A previous study has identified two types of recombinant variants of *Potato virus Y *strain NTN (PVY^NTN^) in China and sequenced the complete genome of the variant PVY^NTN^-HN2. In this study, the complete genome of isolate PVY^NTN^-HN1 was fully sequenced and analyzed. The most striking difference between the two variants was the location of recombinant joint three (RJ3). In PVY^NTN^-HN1, like other typical European-PVY^NTN ^isolates such as PVY^NTN^-Hun, the RJ3 was located at nucleotide (nt) 9183, namely the 3' proximal end of the CP gene (nt. 8571-9371), thus leading to most (the first 613 nucleotides from the 5' proximal end) of the CP gene (801 bp) with a PVY^N ^origin and PVY^N^-serotype; whereas in contrast, the RJ3 in PVY^NTN^-HN2 was located at nt 8572, consequently leading to a CP gene of PVY^O ^origin and PVY^O^-serotype. The varied genome composition among PVY^O^, PVY^N^, PVY^N:O^, PVY^NTN^-HN1 and PVY^NTN^-HN2 made them useful for the investigation of possible roles of gene segment(s) in symptom formation on host plants. When *Physalis floridana *plants were infected with different PVY isolates, two types of symptoms were induced. PVY^N ^and PVY^NTN^-HN1 induced mild symptoms (mainly mild mottling) whereas PVY^O^, PVY^N:O ^and PVY^NTN^-HN2 induced serve symptoms including leaf and stem necrosis, leaf-drop and stunting. These results, together with a previous study using artificial PVY chimeras, demonstrate that the CP gene, especially the 5' proximal segment (nt 8572-9183), and/or CP likely determine the pathogenicity of PVY in *P. floridana*.

## Findings

*Potato virus Y *(PVY) is the type species of the *Potyvirus *genus in the *Potyviridae *family [1]. It infects a number of plant species in the nightshade family (*Solanaceae*) and causes a wide range of symptoms from symptomless to mosaic, mottling, lesions, stunting, necrosis and plant death, depending on the plant species, the cultivar, the virus strain and isolate [1]. PVY possesses a single-stranded positive RNA genome comprised of approximately 9700 nucleotides that encode a polyprotein of approximately 3061 amino acids [2]. The polyprotein undergoes proteolysis to form 10 mature proteins with different functions including replication, transportation and spread of the virus [1,2]. Many strains/substrains of PVY have been recognized according to the primary hosts and host reactions. For the potato-infecting PVY, the ordinary strain (PVY^O^), the tobacco veinal necrosis strain (PVY^N^) and the potato stipple streak strain (PVY^C^) are the first ones to be recognized [1], followed by the potato tuber necrosis strain (PVY^NTN^) and the recombinant N:O/Wilga group (PVY^N:O ^or PVY^N^-Wilga) [3-5]. PVY^NTN ^is characterized by its ability to induce potato tuber necrotic ringspot disease (PTNRD) in sensitive potato cultivars [5-7], whereas PVY^N:O ^is defined by its reaction to PVY^O^-specific antibody (i.e., PVY^O^-serotype) but causing veinal necrosis on tobacco plants (i.e., PVY^N ^pathotype) [5,8]. Two types of PVY^NTN^, one recombinant and the other non-recombinant, have been identified [4,7,9]. The former is represented by PVY^NTN^-Hun [10] and has been referred to as European (Eu)-PVY^NTN ^[7,11-13], and the latter is represented by PVY^NTN^-Tu 660 [7] and has been referred to as North American (NA)-PVY^NTN ^[7,11,12,14]. Both Eu-PVY^NTN ^and NA-PVY^NTN ^react to PVY^N^-specific antibody [11,13]. Recently, a new recombinant PVY^NTN ^variant type has been identified in Syria [15,16] and China [9]. The variant type that includes the isolates PVY^NTN-NW ^[16] and PVY^NTN^-HN2 [9] reacts to PVY^O^-antibody and induces veinal necrosis on tobacco and PTNRD on sensitive potato cultivars [9,16]. Reverse transcription-PCR (RT-PCR) based genotyping has been successfully used to characterize the genome features of the Eu-PVY^NTN^-like isolate PVY^NTN^-HN1 and the PVY^NTN-NW^-like isolate PVY^NTN^-HN2 in China [9]. Here we report the differential responses of *Physalis floridana *to PVY^NTN^-HN1 and PVY^NTN^-HN2 infections. PVY^NTN^-HN1 and PVY^N ^induced mottling on *P. floridana *whereas PVY^NTN^-HN2, PVY^O ^and PVY^N:O ^induced severe symptoms including leaf and stem necrosis, leaf-drop and stunting. The results, together with the genome make-ups of various PVY isolates, suggest that the CP gene plays a significant role in symptom induction in *P. floridana*, consistent with the results reported by Bukovinszki *et al. *[17].

The greenhouse maintained PVY isolates PVY^NTN^-HN1 (formerly PVY sample 1 [9]), PVY^NTN^-HN2, PVY^N^-Jg, PVY^O^-RB and PVY^N:O^-Mb58 in 'Russet Burbank' plants/tubers [5,7,9,11-13] were used in this study. PVY^NTN^-HN1 and PVY^NTN^-HN2 were obtained in China [9], while the rest were from Canada [5,7,11-13]. All isolates have been characterized molecularly by P1 gene- and recombinant joint (RJ)-based RT-PCR assays [12,13], pathologically by tobacco- and potato-based bioassays, and serologically by PVY^O^- and PVY^N^-antibody-based ELISA assays [5,7,9,11-13,18]. Moreover, except for PVY^NTN^-HN1, all of the isolates have been sequenced fully (PVY^NTN^-HN-2, PVY^N^-Jg, PVY^O^-RB) or partially (PVY^N:O^-Mb58) (accession numbers are HM367076, AY166867, GQ200836, AY745493 for PVY^O^-RB, PVY^N^-Jg, PVY^NTN^-HN-2, and PVY^N:O^-Mb58 respectively). To better understand the isolate PVY^NTN^-HN1, especially to reveal the exact nucleotide locations of the recombinant joints that had been detected by RT-PCR [9], the complete genome of PVY^NTN^-HN1 was sequenced. The same nine sets of PCR primers (for primer sequences, see reference [7]) that had been used to clone/sequence various isolates of PVY [5,7,9,18] were used. Each primer pair resulted in a DNA fragment of 1.0 to 1.3 kb, overlapping with adjacent fragments with approximately 100 bp at each end. Each fragment was cloned into a pGM-T cloning vector (TIANGEN Biotech, Beijing, China) according to the manufacturer's instructions; and two clones of each fragment were sequenced from both forward and reverse directions using the universal T7 promoter and SP6-promoter primers at the Sangon Biological Engineering Technology & Services Co. Ltd (Shanghai, China). The complete genome sequence (GenBank accession number HQ631374) was confirmed by re-sequencing overlapping cDNA clones obtained from a separate experiment from RNA isolated from PVY^NTN^-HN1 infected tobacco leaves. Sequence identities were analyzed using BLAST (http://www.ncbi.nlm.nih.gov/BLAST). For detection of the recombinant events, complete nucleotide sequences of various PVY isolates were aligned using ClustalW2 (http://www.ebi.ac.uk/Tools/clustalw2/index.html) [19]. The aligned sequences served as inputs for similarity scanning using the program SimPlot [20, generously provided by the author at http://sray.med.som.jhmi.edu/]. The resulting similarities were plotted along the nucleotide sequences of the virus genome.

**Figure 1:**
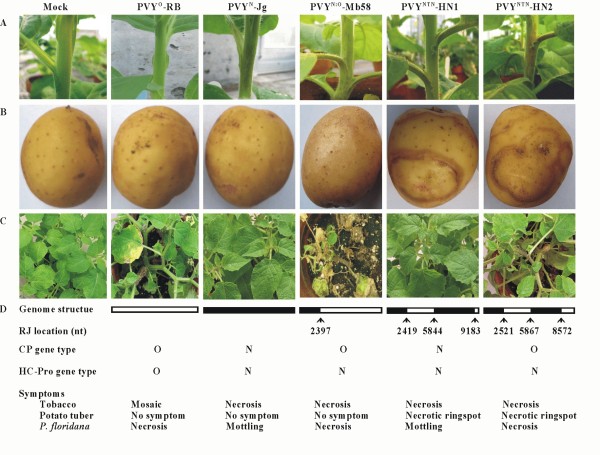
**Symptoms induced by different isolates of *Potato virus Y *(PVY) on tobacco, potato and *Phyalis floridana***. **A**. Symptoms on tobacco (*Nicotiana tabacum *cv. 'Samsun') plants 15 days after inoculation. **B**. Symptoms on potato (*Solanum tuberosum *cv. 'Yukon Gold') tubers harvested from plants 90 days after inoculation. **C**. Symptoms on *P. floridana *25 days after inoculation. **D**. Summary of genome composition, recombinant joint (RJ) locations, CP gene type, HC-Pro gene type and major symptoms of PVY isolates. Isolates used for the experiments are PVY^O^-RB, PVY^N^-Jg, PVY^N:O^-Mb58, PVY^NTN^-HN1 and PVY^NTN^-HN2.

As anticipated, PVY^NTN^-HN1 shared highest sequence identities with PVY^NTN^-Hun, a representative of typical Eu-PVY^NTN^, at both complete nucleotide and polyprotein levels at 99.2% and 99.1%, respectively. It was followed by PVY^NTN^-HN2/PVY^NTN-NW^, PVY^N:O^, PVY^N ^and PVY^O^, represented by isolates PVY^NTN^-HN2, PVY^N:O^-Mb112, PVY^N^-N605 and PVY^O^-RB, respectively (Table 1). The sequence identities between PVY^NTN^-HN1 and PVY^N^-Jg, a NA- PVY^N ^[7,11], were 90.9% and 95.9% at the complete nucleotide and polyprotein levels, respectively (Table 1). As expected, the sequence identities between PVY^NTN^-HN1 and PVY^N:O^-Mb58 (accession number AY745493, partial length) were similar to that between PVY^NTN^-HN1 and PVY^N:O^-Mb112 (data not shown). Further comparison of PVY^NTN^-HN1 with PVY^NTN^-HN2 at mature protein level revealed that the two shared high sequence identities for all proteins (97.8 - 100%) but the CP (Table 1), which was similar to that exhibited in PVY^NTN^-Hun vs PVY^NTN^-HN2 [9]. Sequence screening of PVY^NTN^-HN1 against PVY^O ^(e.g., PVY^O^-RB) and PVY^N ^(e.g., PVY^N^-605 or PVY^N^-Jg) using SimPlot [20] revealed three recombinant joints at nt 2419, 5844 and 9183 in PVY^NTN^-HN1 genome (Figure 1D), resulting from the genome recombination between PVY^N ^and PVY^O^. In contrast, the RJs in PVY^NTN^-HN2 were located at nt 2521, 5867 and 8572 (Figure 1D) [9]. PVY^NTN^-HN1 shares identical RJs with PVY^NTN^-Hun (data not shown). The location of RJ3 in PVY^NTN^-HN1 and PVY^NTN^-Hun at nt 9183, namely the 3' proximal end of CP gene (nt 8571-9371), led to most (the first 613 nt from the 5' proximal end) of the 801-bp-long CP gene of a PVY^N ^origin, which eventually resulted in a PVY^N^-serotype of these isolates [5,7,9]. In contrast, the RJ3 in PVY^NTN^-HN2/PVY^NTN^-SYR-NB-16N (accession number AB270705) at nt 8572, namely the 5'end of the CP gene, led to the complete CP gene of a PVY^O ^origin, which further resulted in a PVY^O^-serotype [9,15]. One RJ, namely RJ1, was present in PVY^N:O ^isolates including PVY^N:O^-Mb112 and PVY^N:O^-Mb58 at nt 2397 [5] (Figure 1D), resulting in a recombinant genome in which the segment prior to the RJ was from PVY^N ^and the remainder from PVY^O ^[5].

**Table 1:** Identities between isolate PVY^NTN^-HN1 and other isolates of *Potato virus Y *(PVY) at both nucleic acid and protein levels

	Length		Sequence Identity (%) (Nucleic acid, Protein)					
	
	Gene nucleotide, size (bp)	Protein size (aa)	**PVY**^**O**^**-RB (HM367076)**	**PVY**^**N**^**-605 (X97895)**	**PVY**^**N**^**-Jg (AY166867)**	**PVY**^**NTN**^**-Hun (M95491)**	**PVY**^**N:O**^**-Mb112 (AY745491)**	**PVY**^**NTN**^**-HN2 (GQ200836)**
5' UTR	1-188, 188	-	66.0, -	100, -	85.6, -	100, -	100, -	99.5, -
P1	189-1013, 825	275	73.0, 71.3	99.3, 99.6	92.2, 91.3	98.7, 98.2	99.6, 100	98.9, 100
HC-Pro	1014-2408, 1395	465	82.2, 90.5	98.8, 99.4	92.6, 96.8	99.1, 99.1	98.4, 99.4	98.4, 98.9
P3	2409-3503, 1095	365	97.3, 98.6	84.7, 92.6	84.6, 92.3	99.4, 99.7	99.1, 99.7	96.3, 97.8
6K1	3504-3659, 156	52	97.4, 100	81.4, 84.6	82.1, 86.5	99.4, 100	100, 100	98.1, 98.1
CI	3660-5561, 1902	634	97.3, 99.1	84.1, 95.7	84.1, 95.7	99.6, 99.5	99.3, 99.5	98.7, 99.7
6K2	5562-5717, 156	52	95.5, 100	82.1, 90.4	79.5, 90.4	100, 100	100, 100	99.4, 98.1
VPG	5718-6281, 564	188	86.9, 92.0	97.0, 99.5	95.6, 97.3	98.9, 99.5	87.8, 94.7	97.9, 99.5
NIa	6282-7013, 732	244	80.9, 93.6	99.3, 98.2	97.0, 98.8	99.2, 98.8	80.6, 92.2	99.0, 99.2
NIb	7014-8570, 1557	519	83.5, 93.6	98.8, 99.0	97.6, 98.7	98.7, 98.7	84.9, 94.4	98.2, 99.0
CP	8571-9371, 801	267	90.8, 92.9	97.5, 98.9	96.5, 98.9	99.4, 98.9	91.4, 94.4	91.6, 94.0
3' UTR	9372-9702, 331	-	98.2, -	84.0, -	85.8, -	99.1, -	98.8, -	99.1, -
Full length	1-9702, 9702	3061	87.9, 92.8	93.2, 97.3	90.9, 95.9	99.2, 99.1	94.1, 97.4	97.7, 98.7

Previous study has revealed the pathotypes of PVY^NTN^-HN1 and PVY^NTN^-HN2 on tobacco and potato [9]. To further characterize the biological properties of these isolates, and, moreover, to investigate whether the different RJ3 sites, namely different CP gene types, play a role in symptom induction in different plant species, tobacco (cv. 'Samsun'), potato (cv. 'Yukon Gold') and *Physalis floridana *plants were mechanically inoculated with PVY^NTN^-HN1, PVY^NTN^-HN2, PVY^N^-Jg, PVY^O^-RB, and PVY^N:O^-Mb58 as described previously [9]. Mock (buffer)-inoculated plants were used as a healthy control. As shown in Figure 1A, petiole and stem necrosis occurred on tobacco plants 15 days after inoculation with PVY^N^-Jg, PVY^N:O^-Mb58, PVY^NTN^-HN1 or PVY^NTN^-HN-2. Veinal necrosis also developed on these plants. On the other hand, the PVY^O^-infected plants only developed mosaic symptoms on the leaves and were free of veinal, petiole and stem necrosis (Figure 1A). No symptoms were observed on the mock-inoculated plants. Various studies have indicated that HC-Pro plays an important role in necrosis development on tobacco plants [5,21,22]. All isolates but PVY^O^-RB possessed a PVY^N^-type of HC-Pro gene [5,7,9,18] (Figure 1D), and therefore induced PVY^N^-like symptoms including veinal/petiole/stem necrosis on tobacco plants. When inoculated to 'Yukon Gold' plantlets (5-leaf-stage), the isolates induced varied foliar symptoms including mild mottling (PVY^N^-Jg), mosaic (PVY^N:O^-Mb58) and severe mosaic/stunting/leaf deformation (PVY^O^-RB, PVY^NTN^-HN1 and PVY^NTN^-HN2) (data not shown), consistent with the previous report [9]. No visible symptoms were observed on potato tubers produced from plants infected with PVY^N^-Jg, PVY^O^-RB or PVY^N:O^-Mb58; and in contrast, distinct necrotic ringspots were observed on potato tubers harvested from plants infected with PVY^NTN^-HN1 or PVY^NTN^-HN2 (Figure 1B), thus confirming that both types of recombinant PVY^NTN ^isolates are capable of inducing PTNRD in sensitive potato cultivars.

It has been known that PVY^O ^induces necrosis in *Physalis floridana*, whereas PVY^N ^incites mottling in this species [1]. Using N/O hybrids comprised of the chimeric genome of PVY^N^-N605 [23] and PVY^O^, the symptom formation on *P. floridana *due to PVY infection was mapped to the CP gene region [17]. Because of the varied genome compositions among the isolates (Figure 1D), they could be used to investigate the putative role of genome segment(s) of PVY in symptom development on *P. floridana*, as done on tobacco [5,24]. Severe symptoms including leaf and stem necrosis, leaf-drop and stunting were observed on *P. floridana *plants infected with PVY^O^-RB, PVY^N:O^-Mb58 and PVY^NTN^-HN2 three weeks after inoculation (Figure 1C), and as time progressed, the symptoms became more distinct. The isolate PVY^N:O^-Mb58 led to plant death five weeks after the inoculation. On the other hand, mild symptoms, mainly mottling, were observed on PVY^N^-Jg and PVY^NTN^-HN1 infected *P. floridana *plants (Figure 1C). Taken together, it can be concluded that the CP gene originated from PVY^O ^is likely responsible for the severe symptoms in PVY^O^-, PVY^N:O^- or PVY^NTN^-HN2-infected *P. floridana *plants. These results, together with the results obtained using artificial PVY chimeras [17], demonstrate that the CP gene, especially the 5' proximal segment (nt 8572-9183) of the gene, plays a critical role in symptom formation in *P. floridana *upon PVY infection, and determines the pathoginicity of PVY isolates. The 3' proximal segment of NIb gene (nt 8136-8570) does not appear to be involved in the symptom formation in *P. floridana *as suggested by Bukovinszki *et al. *[17]. It is also noteworthy that the different symptoms incited by different PVY types/isolates in tobacco, potato and *P. floridana *can be used to uncover the genome compositions of the virus.

## Competing interests

The authors declare that they have no competing interests.

## Authors' contributions

XH carried out the experiments. XN designed, analyzed and wrote the paper. HC and XX collected isolates PVY^NTN^-HN1 and PVY^NTN^-HN2, participated in experiment planning and execution. All authors read and approved the final manuscript.

## References

[B1] Shukla DD Ward CW Brunt AA The Potyviridae 1994 Wallingford, Oxon, UK: CAB International

[B2] Riechmann JL Laín S García JA Highlights and prospects of potyvirus molecular biology J Gen Virol 1992 73 1 16 10.1099/0022-1317-73-1-1 1730931

[B3] Chrzanowska M New isolates of the necrotic strain of potato virus Y (PVY^N^) found recently in Poland Potato Res 1991 34 178 182

[B4] Glais L Tribodet M Kerlan C Genomic variability in *Potato potyvirus Y *(PVY): evidence that PVY^N^W and PVY^NTN ^variants are single to multiple recombinants between PVY^O ^and PVY^N ^isolates Arch Virol 2002 147 363 378 10.1007/s705-002-8325-0 11890528

[B5] Nie X Singh RP Singh M Molecular and pathological characterization of N:O isolates of the *Potato virus Y *from Manitoba, Canada Can J Plant Pathol 2004 26 573 583 10.1080/07060660409507178

[B6] Le Romancer M Kerlan C Nedellec M Biological characterization of various geographical isolates of potato virus Y inducing superficial necrosis on potato tubers Plant Pathol 1994 43 138 144 10.1111/j.1365-3059.1994.tb00563.x

[B7] Nie X Singh RP Evolution of North American PVY^NTN ^strain Tu 660 from local PVY^N ^by mutation rather than recombination Virus Genes 2003 26 39 47 10.1023/A:1022326021195 12680692

[B8] Singh RP McLaren DL Nie X Singh M Possible escape of a recombinant isolate of *Potato virus Y *by serological indexing and methods of its detection Plant Dis 2003 87 679 685 10.1094/PDIS.2003.87.6.679 30812860

[B9] Hu X He C Xiao Y Xiong X Nie X Molecular characterization and detection of recombinant isolates of *Potato virus Y *from China Arch Virol 2009 154 1303 1312 10.1007/s00705-009-0448-z 19597695

[B10] Thole V Dalmay T Burgyan J Balazs E Cloning and sequencing of potato virus Y (Hungarian isolate) genomic RNA Gene 1993 123 149 156 10.1016/0378-1119(93)90118-M 8428653

[B11] Nie X Singh RP Probable geographical grouping of PVY^N ^and PVY^NTN ^based on sequence variation in P1 and 5'-UTR of PVY genome and methods for differentiating North American PVY^NTN^ J Virol Methods 2002 103 145 156 10.1016/S0166-0934(02)00023-X 12008009

[B12] Nie X Singh RP A new approach for the simultaneous differentiation of biological and geographical strains of *Potato virus Y *by uniplex and multiplex RT-PCR J Virol Methods 2002 104 41 54 10.1016/S0166-0934(02)00037-X 12020791

[B13] Nie X Singh RP Specific differentiation of recombinant PVY^N:O ^and PVY^NTN ^strains by multiplex RT-PCR J Virol Methods 2003 113 69 77 10.1016/S0166-0934(03)00221-0 14553892

[B14] Piche LM Singh RP Nie X Gudmestad NC Diversity among Potato virus Y isolates obtained from potatoes grown in the United States Phytopathology 2004 94 1368 1375 10.1094/PHYTO.2004.94.12.1368 18943708

[B15] Chikh Ali M Maoka T Natsuaki KT The occurrence and characterization of new recombinant isolates of PVY displaying shared properties of PVY^NW ^and PVY^NTN^ J Phytopath 2007 155 409 415 2007 10.1111/j.1439-0434.2007.01251.x

[B16] Chikh Ali M Maokac T Natsuakib T Natsuaki KT PVY^NTN-NW^, a novel recombinant strain of *Potato virus Y *predominating in potato fields in Syria Plant Pathol 2010 59 31 41 10.1111/j.1365-3059.2009.02174.x

[B17] Bukovinszki A Gotz R Johansen E Maiss E Balazs E The role of the coat protein region in symptom formation on *Physalis floridana *varies between PVY strains Virus Res 2007 127 122 125 10.1016/j.virusres.2007.03.023 17482305

[B18] Nie B Singh M Sullivan A Singh RP Xie C Nie X Recognition and molecular discrimination of severe and mild PVY^O ^variants of *Potato virus Y *in potatoes in New Brunswick, Canada Plant Dis 2011 95 113 119 10.1094/PDIS-04-10-0257 30743422

[B19] Larkin MA Blackshields G Brown NP Chenna R McGettigan PA McWilliam H Valentin F Wallace IM Wilm A Lopez R Thompson JD Gibson TJ Higgins DG ClustalW and ClustalX version 2 Bioinformatics 2007 23 2947 2948 10.1093/bioinformatics/btm404 17846036

[B20] Lole KS Bollinger RC Paranjape RS Gadkari D Kulkarni SS Novak NG Ingersoll R Sheppard HW Ray SC Full-length human immunodeficiency virus type 1 genomes from subtype C-infected seroconverters in India, with evidence of intersubtype recombination J Virol 1999 73 152 160 9847317 10.1128/jvi.73.1.152-160.1999 PMC103818

[B21] Tribodet M Glais L Kerlan C Jacquot E Characterization of *Potato virus Y *(PVY) molecular determinants involved in the vein necrosis symptom induced by PVY^N ^isolates in infected *Nicotiana tabacum *cv. Xanthi J Gen Virol 2005 86 2101 2105 10.1099/vir.0.80926-0 15958688

[B22] Hu X Meacham T Ewing L Gray SM Karasev AV A novel recombinant strain of *Potato virus Y *suggests a new viral genetic determinant of vein necrosis in tobacco Virus Res 2009 143 68 76 10.1016/j.virusres.2009.03.008 19463723

[B23] Jakab G Droz E Brigneti G Baulcombe D Malnoe P Infectious in vivo and in vitro transcripts from a full-length cDNA clone of PVY-N605, a Swiss necrotic isolate of potato virus Y J Gen Virol 1997 78 3141 3145 9400962 10.1099/0022-1317-78-12-3141

[B24] Ramírez-Rodríguez V Aviña-Padilla K Frías-Treviño G Silva-Rosales L Martínez-Soriano J Presence of necrotic strains of *Potato virus Y *in Mexican potatoes Virology J 2009 6 48 10.1186/1743-422X-6-48 PMC2704195 19419565

